# Years of Blindness Lead to “Visualize” Space Through Time

**DOI:** 10.3389/fnins.2020.00812

**Published:** 2020-08-04

**Authors:** Maria Bianca Amadeo, Claudio Campus, Monica Gori

**Affiliations:** ^1^Unit for Visually Impaired People, Istituto Italiano di Tecnologia, Genova, Italy; ^2^Department of Informatics, Bioengineering, Robotics and Systems Engineering, Università degli Studi di Genova, Genova, Italy

**Keywords:** spatial perception, temporal perception, late blindness, EEG, auditory processing

## Abstract

Spatial representation has been widely studied in early blindness, whereas research about late blindness is still limited. We recently demonstrated that the early (50–90 ms) event-related potential (ERP) response observed in sighted people during a spatial bisection task, is altered in early blind people and is influenced by the amount of time spent without vision in late blind individuals. Specifically, in late blind people a shorter period of blindness is associated with strong contralateral activation in occipital cortex and good performance during the spatial task–similar to that of sighted people. In contrast, non-lateralized occipital activation and lower performance characterize late blind individuals who have experienced a longer period of blindness–similar to that of early blind people. However, the same early occipital response activated in sighted individuals by spatial cues has been found to be activated by temporal cues in early blind individuals. Here, we investigate whether a similar temporal attraction can explain the neural and behavioral changes observed after many years of blindness in late blind people. An EEG recording was taken during a spatial bisection task where coherent and conflicting spatio-temporal information was presented. In participants with long blindness duration, the early recruitment of both visual and auditory areas is sensitive to temporal instead of spatial coordinates. These findings highlight some limits of neuroplasticity. Perceptual advantages from cross-sensory calibration during development seem to be subsequently lost following years of visual deprivation. This result has important implications for clinical outcomes following late blindness, highlighting the importance of timing in intervention and rehabilitation programs that activate compensatory strategies soon after sensory loss.

## Introduction

From birth, infants gradually learn to combine the spatial information arriving from their sensory modalities into a coherent multisensory representation of space (Bremner et al., 2008). The visual modality has a fundamental role in this process, making it possible to obtain an instant and exhaustive representation of the surrounding environment in a single frame (Tinti et al., 2006). Given the importance of visual experience, what happens to spatial representation when visual input is missing is a key question (e.g., Ricciardi et al., 2020).

Spatial representation has been extensively studied in early blindness (for a review, Voss, 2016). Studies reveal conflicting results about spatial performance following early visual loss. On the one hand, blindness can lead to the functional recruitment of visual areas and to enhance the remaining senses (e.g., Gougoux et al., 2004). On the other hand, studies suggest that the lack of visual input negatively affects some additional spatial processing (e.g., Gori et al., 2014). The study of late blindness has often been neglected (for a review, Voss, 2013). Since late blind (LB) people experience visual calibration in childhood and blindness in adulthood, study of their spatial reasoning can deepen our knowledge about the role of visual experience and deprivation on the way in which the brain builds spatial representations. The intersection of visual experience and deprivation leads to peculiar spatial skills and neural correlates, in some cases similar to those of sighted people (e.g., Wanet and Veraart, 1985; Finocchietti et al., 2015b) and in others resembling those of early blind individuals (e.g., Voss et al., 2004; Fieger et al., 2006).

A consistent body of literature on late blindness has focused on the age of blindness onset to investigate critical time windows where visual experience is necessary to develop specific abilities (e.g., Li et al., 2013, 2016). Interestingly, we have recently demonstrated that, in a complex spatial task, years of blindness matter more than the age of onset (Amadeo et al., 2019). Specifically, we observed that neural activation associated with the behavioral performance of LB people during a spatial bisection task was influenced by the amount of time spent without vision (or, blindness duration, BD). The spatial bisection task consists of listening to a sequence of three sounds and judging the relative spatial position of the second sound with respect to the other two sounds. The second sound is crucial to perform the bisection task because it represents the initial point for the construction of a spatial metric. We observed that immediately after vision loss, performance and neural correlates of LB individuals are similar to those of sighted people. They succeed in the task, and exhibit the same early (50–90 ms) contralateral activation observed in the occipital areas of sighted individuals after the second sound of the spatial bisection (Campus et al., 2017). Yet, with years of visual deprivation, spatial bisection skills and neural correlates of LB people become modified. Specifically, after more than 25 years of BD, LB individuals show a weaker and non-lateralized occipital response to the second sound, and an associated lower performance. The same neural pattern of response has been previously observed in early blind people, who are unable to perform the task (Campus et al., 2019).

Furthermore, when early visual experience is lacking, people use a different strategy to represent complex configurations of space (Gori et al., 2018, 2020b). In fact, early blind people are strongly influenced by temporal representations of events when inferring auditory spatial representations. Manipulating spatial and temporal coordinates of sounds during a spatial bisection task reveals this. We created conditions of coherence between space and time by associating a short/long spatial distance between two sounds with a short/long temporal interval between them. With spatiotemporal coherence, the spatial bisection deficit of early blind people disappeared. Moreover, under these conditions, the same early contralateral occipital response observed in sighted individuals was present. Thus, occipital activation selective for the spatial position of the second sound in the spatial bisection task is observed in early blind individuals when temporal cues are informative about space. We also created conditions of conflict between space and time associating a short spatial distance between sounds with a long temporal interval between them, and similarly a long spatial distance with a short temporal interval. When conflicting spatiotemporal information was presented, the behavioral deficit of early blind people increased. Further, at the cortical level, while the early contralateral occipital response was still present in early blind people, its topography was inverted. Namely, the topography appeared based on the virtual position of the second stimulus as defined by its temporal delay. Interestingly, the auditory cortical response of early blind individuals was similarly contralateral to the position of the second sound as indicated by the temporal delay. These results suggest that the same circuits responding to spatial cues in sighted individuals may be sensitive to temporal cues following early visual deprivation. Audition is the most reliable sense for temporal representation (e.g., Burr et al., 2009). It could be that when the visual calibration of the auditory space is missing during childhood (see cross-calibration theory, Burr and Gori, 2012), the auditory modality strongly adheres to the temporal domain. This could result in auditory spatial maps based on a temporal coordinate system when visual experience is missing.

Given that LB individuals with a long BD closely resemble early blind individuals in the spatial bisection task, we tested whether a similar temporal dominance could explain the neural and behavioral changes driven by years of blindness in LB individuals. To this end, we replicated the experimental paradigm previously performed with early blind people with LB participants with different years of BD. Electroencephalographic (EEG) and psychophysical responses were recorded during a spatial bisection task when coherent and conflicting spatiotemporal cues were delivered. As in previous studies (Amadeo et al., 2019; Campus et al., 2019), we performed a temporal bisection task as a control experiment, where the subject evaluated temporal intervals instead of spatial distances between three sounds. Results suggest that BD in LB individuals is associated with a tendency to build spatial maps relying on temporal information during the spatial bisection task.

## Materials and Methods

### Participants

The sample consisted of 12 late-onset (LB) blind individuals [mean age ± standard deviation (*SD*): 50.25 ± 15.85 years; females = 3, see Table 1 for clinical details], and 12 blindfolded sighted (S) individuals [48.52 ± 13.56 years; *t*-test for age: *t*(21.5) = −0.33, *p* = 0.7]. Age of blindness onset ranged from 6 to 51 (24.75 ± 15.82) years of age, and BD ranged from 5 to 54 (25.5 ± 15.29) years. All blind subjects were completely blind and lacked hearing problems (this was verified prior to testing). Participants involved in the study were the same LB and sighted individuals that took part in our previous experiment (see Amadeo et al., 2019). Written informed consent was required prior to participation. The experiment was conducted in accordance with the Declaration of Helsinki, after ethics approval from the local health committee (Comitato Etico Regione Liguria).

**TABLE 1:** Clinical details of the late blind participants (*N* = 12).

Participant	Age	Gender	Pathology	Blindness onset	Blindness duration
S1	26	M	Leber amaurosis	13	13
S2	26	F	Glaucoma	6	20
S3	29	M	Corneal opacity	17	12
S4	45	M	Glaucoma	6	39
S5	49	M	Retinis Pigmentosa	40	9
S6	51	F	Leber amaurosis	46	5
S7	54	M	Chiasmatic glioma	14	40
S8	58	M	Glaucoma	20	38
S9	65	M	Retinis Pigmentosa	38	27
S10	65	F	Retinis Pigmentosa	32	33
S11	67	M	Retinal detachment	51	16
S12	68	M	Glaucoma	14	54

*For each participant, data from left to right are chronological age, gender, pathology, age of blindness onset, and years of blindness duration (i.e., number of years spent without vision).*

### Stimuli and Procedure

**FIGURE 1:**
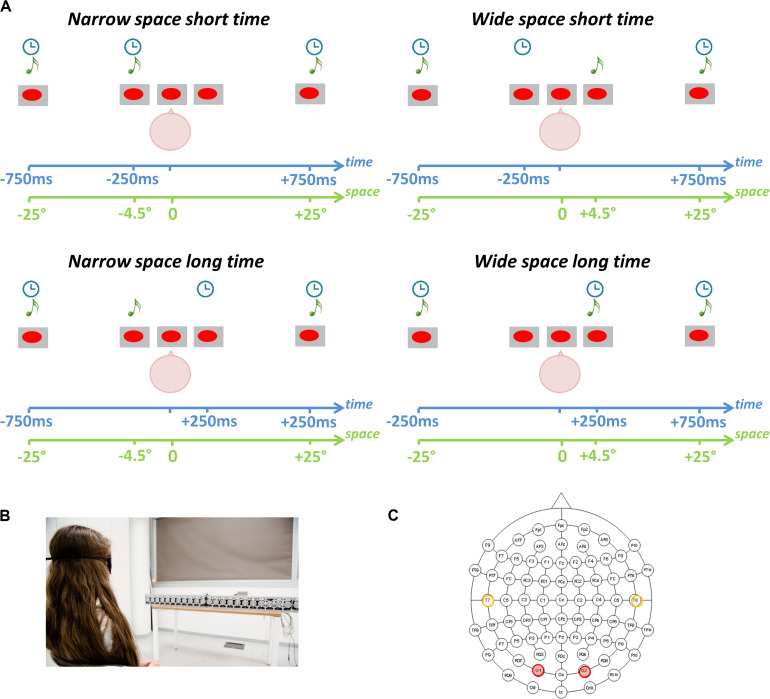
Spatial and temporal bisection tasks. **(A)** Experimental paradigm. Three sounds (S1, S2, S3) were played from three spatial positions and at three different time points. Participants evaluated the relative position of S2 in space (spatial bisection) or time (temporal bisection) with respect to the other two sounds (S1 and S3). Stating that 0° represents the central speaker, S1 was always played from -25° (i.e., left) while S3 was always played from + 25° (i.e., right). Stating that 0 ms represents the halfway point of the trial duration, S1 was always played at -750 ms while S3 was always played at + 750 ms. Based on the condition, S2 was played randomly and independently from ± 4.5° in space and at ± 250 ms. The interaction of spatial and temporal coordinates of S2 leads to four conditions: (i) *narrowSpace_shortTime*: S2 from -4.50° at -250 ms (Top Left); (ii) *narrowSpace_longTime*: S2 from -4.50° at + 250 ms (Bottom Left); (iii) *wideSpace_shortTime*: S2 from + 4.50 at −250 ms (Top Right); (iv) *wideSpace_longTime*: S2 from + 4.50 at + 250 ms (Bottom Right). **(B)** Setup. Participants sat in front of free-field speakers. **(C)** Electrode montage. EEG analyses focused on left (T7) and right (T8) temporal electrodes (orange) and left (O1) and right (O2) occipital electrodes (red).

Participants performed a spatial and a temporal bisection task. They sat in front of a set of free-field speakers placed in the lower visual hemifield. Three stimuli were played at three different spatial positions (Figure 1A) and times (Figure 1B). Stimuli consisted of sounds with the following characteristics: 500 Hz, 75 ms duration, 60 dB SPL at the subject position. The first sound (S1) was delivered at -25°, while the third sound (S3) was delivered at + 25° (with 0° representing the central speaker, negative values on the left and positive values on the right). The temporal interval between S1 and S3 was fixed at 1.5 s. The second sound (S2) could be played from either -4.5° (left) or 4.5° (right; Figure 1A) in space, and independently at either -250 ms or + 250 ms in time (Figure 1B; with 0 ms representing the middle of the temporal sequence). These values were chosen based on previous literature (e.g., Amadeo et al., 2019; Campus et al., 2019). The task consisted of evaluating either the spatial distances (spatial bisection) or the temporal intervals (temporal bisection) between the three sounds. Specifically, participants had to answer if the distance/interval between S1 and S2 (i.e., the first distance/interval) was smaller or larger than the distance/interval between S2 and S3 (i.e., the second distance/interval). Presentation order of the spatial and temporal bisection tasks was randomized between subjects. A trial with S2 played from the left (-4.5°) side of the subject (a smaller first distance) is referred to as “narrow” space, while that with S2 played from the right (+ 4.5°) side of the subject (a larger first distance) is referred to as “wide” space. Similarly, S2 played sooner (-250 m) is referred to as “short” time, while S2 played later (+ 250 ms) is referred to as “long” time.

Thus, four conditions were possible: (1) S2 from -4.50° at -250 ms (i.e., *narrowSpace_shortTime*: first distance/interval narrow in space and short in time), (2) S2 from -4.50° at + 250 ms (i.e., *narrowSpace_longTime*: first distance/interval narrow in space and long in time), (3) S2 from + 4.50° at -250 ms (i.e., *wideSpace_shortTime*: first distance/interval wide in space and short in time), and (4) S2 from + 4.50° at + 250 ms (i.e., *wideSpace_longTime*: first distance/interval wide in space and long in time). Exactly the same stimuli were used in the temporal and spatial bisection tasks, and each bisection task consisted of 60 trials per condition (i.e., 240 trials). An S2 was also delivered at 0° and at 0 ms during catch trials (i.e., *equalSpace_equalTime*; number of catch trials = 15). Inter-trial interval was 1250 ± 250 ms. Subjects were instructed to answer by pressing the appropriate button when all the three sounds were delivered, i.e., after S3. The time employed to answer was recorded to ensure participants were engaged in the task. For more information about setup and procedure refer to Gori et al. (2020b).

### EEG Data Collection and Preprocessing

We recorded high-density EEG from 64 scalp electrodes with the Biosemi ActiveTwo EEG System (Figure 1B). Data were acquired at 2048 Hz and then downsampled to 512 Hz after band pass filtering from DC to 134 Hz. Two additional electrodes were used (left/right outer canthi) to check ocular movements. The EEG was filtered between 0.1 and 100 Hz. To remove stereotypical and non-stereotypical transient high-amplitude artifacts, we applied the Artifact Subspace Reconstruction technique implemented by the EEGLAB plug-in (Delorme and Makeig, 2004; Mullen et al., 2013). We kept all parameters at default values except that we used a sliding window of 500 ms. Independent Component Analysis was used to clean the EEG data (Delorme and Makeig, 2004); specifically, we used SASICA (Chaumon et al., 2015) and IC_MARC (Frølich et al., 2015), two EEGLAB toolboxes. We kept all parameters at their default and referred to validation papers for component rejection. We used the mean of left and right mastoids as reference. For more information about EEG data processing refer to Gori et al. (2020b).

### Behavioral and EEG Data Analysis

We wished to test if, with increasing BD, temporal cues during a spatial bisection task alter performance and recruitment of the visual and auditory cortices of LB individuals in a manner similar to that seen in early blind individuals (Gori et al., 2020b). In fact, we previously showed that in early blind people the second sound (S2) of the spatial bisection does not produce the early contralateral occipital activation observed in sighted individuals (Campus et al., 2019). A similar pattern characterizes LB subjects that spent many years without seeing (Amadeo et al., 2019), but not LB subjects that recently lost sight. The lack of this response has been proposed as the neural correlate of a low performance at the task. However, by adding coherent or conflicting temporal cues in the spatial bisection task, we noticed that, in early blind people, the same early occipital response is elicited by temporal cues (Gori et al., 2020b). To test whether the same mechanism characterizes performance and cortical recruitment of LB people with long blindness duration (BD), we added coherent or conflicting temporal cues to the spatial bisection task and focused EEG analyses on the early cortical responses to S2 of the spatial bisection task. We used the responses to S2 during a temporal bisection task as a control to verify that the mechanism is specific to the spatial task.

First of all, statistical analyses were conducted to investigate differences in the behavioral performance (i.e., percentage of correct responses) between S and LB groups in the spatial and temporal bisection task. Prior to perform analyses, logit transformation was applied to percentage of correct responses. For each task (i.e., separately for spatial and temporal bisection tasks), conditions were grouped based on the congruence or incongruence of the spatiotemporal coordinates of S2. This led to two merged conditions: coherent trials (*narrowSpace_shortTime* and *wideSpace_longTime*), and conflicting trials (*narrowSpace_ longTime* and w*ideSpace_shortTime*). For each bisection task (spatial and temporal), comparisons between percentage of correct responses were done using a two-way ANOVA, with group (S,LB) as a between-subjects factor, and condition (coherent, conflicting) as a within-subjects factor. *Post hoc* comparisons were conducted using two-tailed t-tests, with probabilities treated as significant when lower than 0.05 after Bonferroni correction. If temporal information helps LB people as hypothesized, their performance should be higher in the coherent conditions where temporal cues can be used to correctly perform the task.

At a neurophysiological level, EEG data were averaged encompassing S2 onset. To obtain event-related potentials (ERPs), we used as baseline a period of 200 ms before the beginning of each trial. After artifact removals, we required a minimum of 40 trials for each of the four spatial and temporal conditions. Catch trials were excluded from statistical analyses. For each ERP the total number of trials was equal to 1410, approximately 59 per subject. Based on our hypothesis (Gori et al., 2020b), we focused our analyses on a time window of 50–90 ms after the sounds, using electrodes involved in visual (O1, O2 in occipital areas) and auditory (T7, T8 in temporal areas) activity (Figure 1C). The choice of time window and scalp sites was based on microstate analyses and topographic analysis of covariance conducted in a previous study of spatial bisection skills following late blindness (Amadeo et al., 2019). In addition, we knew from previous studies that spatial bisection skills are reflected by a specific ERP component in the time window between 50 and 90 ms after the second sound (S2) of the task, as well as contralateral occipital electrodes (Campus et al., 2017). To obtain mean ERP amplitude, we averaged the voltage in the selected (50–90 ms) time window.

We focused on spatial performance and neural correlates of LB participants to investigate the influence of BD years. Since we were interested in how the subject’s responses related to the stimuli presented, instead of analyzing overall percentage of correct responses in merged conditions (coherent, conflicting), we analyzed the percentage of trials in which the subject perceived the first distance as wider for each condition (i.e., *narrowSpace_shortTime*, *wideSpace_longTime, narrowSpace_longTime*, *wideSpace_shortTime*). For coherent conditions, a narrow (i.e., S2 delivered from the left) and wide (i.e., S2 delivered from the right) first distance in space corresponded to a short and long first interval in time respectively. However, for conflicting conditions, a narrow and wide first distance in space corresponded to a long and short first interval in time respectively. Therefore, in the conflicting conditions, S2 could be physically delivered from the left (i.e., closer to S1) but temporally closer to S3 (i.e., right; *narrowSpace_longTime*). Alternatively, S2 could be physically delivered from the right (i.e., closer to S3) but temporally closer to S1 (i.e., left; *wideSpace_shortTime*). If participants use the temporal information to perform the task, their performance in the conflicting conditions should be based on the virtual position of the second sound, as indicated by the temporal delay rather than actual spatial distance. Since our hypothesis was that after many years of blindness LB people are attracted by the temporal information, we conducted an analysis of covariance (ANCOVA) with: percentage of trials (logit-transformed) in which subject perceived the first distance as wider as the dependent variable, condition as a factor and BD and age of onset as covariates. Based on literature (e.g., Li et al., 2013, 2016), we decided to include age of onset as a covariate to rule out an effect of this variable on results. Based on our prediction, we expected a significant interaction only between condition and BD. Specifically, during the conflicting conditions, with increasing BD, participants should perceive the first distance as wider when the first interval is longer in time, and actually narrower in space. On the other hand, BD should not influence responses in the coherent conditions where spatial and temporal cues are congruent. Thus, for each condition, we subsequently carried out *post hoc* linear regressions between percentage of trials in which the subject perceived the first distance as wide and BD.

Since the neural correlates of spatial bisection skills are well established, we used a similar approach to investigate whether BD influences the ERP response in occipital and temporal areas during the conflicting conditions. For each electrode (O1, O2, T8, T9), we built an ANCOVA with individual mean ERP amplitude in the selected time window as the dependent variable, condition (*narrowSpace_shortTime*, *wideSpace_longTime, narrowSpace_longTime*, *wideSpace_shortTime*) as a factor, and BD and age of onset as covariates. Again, we included age of onset in to account for the possibility that results could be related to this variable. Subsequently, for each electrode and condition of the spatial bisection task, we performed *post hoc* linear regressions between individual mean ERP amplitude in the 50–90 ms time window and years of BD. Indeed, if after many years of blindness individuals use temporal cues to evaluate spatial distances, the ERP response to S2 in the conflicting conditions (*narrowSpace_longTime*, *wideSpace_shortTime*) should gradually invert its topography. For the sake of clarity, we here explicitly predict results based on the case that with increasing BD, people start to answer using the virtual position of S2 suggested by the temporal delay. Given that typically a cortical response is more contralateral to the physical position of a sound, in the condition *narrowSpace_longTime* (i.e., S2 spatially from the left but temporally closer to S3, which is played from the right), the response in O1 and T7 (ipsilateral to the physical spatial position, but contralateral to the virtual position suggested by the temporal delay of the sound) should increase with BD, while the response in O2 and T8 (contralateral to the physical spatial position, but ipsilateral to the virtual position suggested by the temporal delay of the sound) should decrease. Conversely, in the condition *wideSpace_shortTime* (i.e., S2 spatially from the right but temporally closer to S1, which is played from left), the response in O1 and T7 should decrease with BD, while the response in O2 and T8 should increase. Summarizing, we did not expect any effect of BD in the coherent conditions, as the spatial position and temporal delay give congruent information and all participants should be able to perform the task. Thus, only in the conflicting conditions do we expect that with increasing BD, electrodes physically contralateral to the real spatial position of sounds attenuate their response, while those contralateral to the virtual position suggested by the temporal cues show a higher activation.

Scalp topographies of mean ERP amplitude in the 50–90 ms time window were evaluated for each condition of spatial and temporal bisection tasks. Since BD linearly affects neural circuits associated with spatial bisection skills (see also Amadeo et al., 2019), for illustrative purposes only, the median BD (23.5 years) was arbitrarily used to split the sample to visualize the different neural activation between those who had been blind for a shorter period of time (i.e., short BD) and those who had been blind for many years (i.e., long BD). The same approach was used to graphically represent ERPs elicited by S2 at occipital (O1, O2) and temporal (T7, T8) electrodes during the spatial bisection task. To further exclude a role of chronological age on results, we run a linear regression analysis to investigate the association between years of blindness and biological age of LB participants.

## Results

Overall, results showed that, with increasing blindness duration (BD) years, temporal cues alter performance and recruitment of the visual and auditory cortices of LB individuals during a spatial bisection task. We previously showed the second sound (S2) of the spatial bisection produces an early contralateral occipital activation in sighted individuals but not in early blind people (Campus et al., 2019) or LB people with long BD (Amadeo et al., 2019). However, by adding coherent temporal cues, the same early occipital response is elicited in early blind people (Gori et al., 2020b). Here, we tested whether coherent temporal cues influence performance and cortical recruitment of LB people with long BD.

### Behavioral Differences in Performance

**FIGURE 2:**
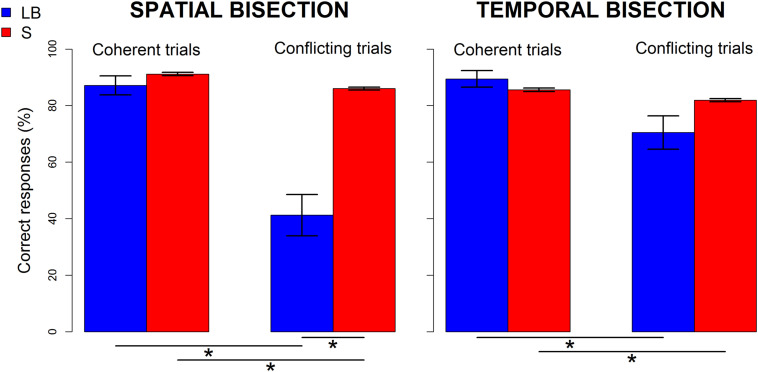
Performance of late blind (LB, blue) and sighted (S,red) individuals in spatial **(left)** and temporal **(right)** bisection tasks. Percentage of correct responses before logit transformation (mean ± SEM) is shown separately for coherent (i.e., *narrowSpace_shortTime* and *wideSpace_longTime*) and conflicting trials (i.e., *narrowSpace_longTime, wideSpace_shortTime*). Stars indicate significant differences (*p* < 0.01).

Behavioral differences in performance (i.e., percentage of correct responses after logit transformation) between groups showed a strongly significant interaction between group (LB, S) and condition (coherence, conflict) for the spatial [*F*(1, 22) = 15.55, *p* < 0.0001, ges = 0.25] but not the temporal [*F*(1, 22) = 4.47, *p* = 0.05, ges = 0.06] bisection task (Figure 2). For the spatial bisection task (Figure 2 left), the performance of LB participants in conflicting trials is significantly lower than that of sighted people [*t*(11.2) = -4.04, *p* = 0.004], and their own performance in coherent trials [*t*(11) = 4.7, *p* = 0.001]. Although performance of sighted people decreased in conflicting compared to coherent conditions [*t*(11) = 23.5, *p* < 0.001], their performance was always well above chance (i.e., > 75%, mean ± standard error of the mean (SEM), for coherent trials: 91 ± 0.6%; for conflicting trials: 86 ± 0.5%). In contrast, the performance of LB people in conflicting conditions was drastically reduced (for coherent trials: 87 ± 3%; for conflicting trials: 41 ± 7%). For the temporal bisection task (Figure 2 right), only a main effect of condition was significant [*F*(1, 22) = 11.02, *p* = 0.003, ges = 0.14], reflecting a slight decrease in performance during the conflicting conditions for both groups [for LB: coherent trials: 89.4 ± 3%, conflicting trials: 70.5 ± 6%, *t*(11) = 2.7, p = 0.04; for S: coherent trials: 85.6 ± 0.6%, conflicting trials: 81.9 ± 0.5%, *t*(11) = 14.3, *p* < 0.001]. Thus, behavioral results suggest that LB individuals are specifically sensitive to the spatiotemporal conflicts during spatial judgments and improve their spatial performance when temporal information is aligned with spatial information. In contrast, the cross-domain conflict or coherence during temporal judgments had a similar, negligible influence in both groups.

### Impact of Years of Blindness on Spatial Bisection Performance

**FIGURE 3:**
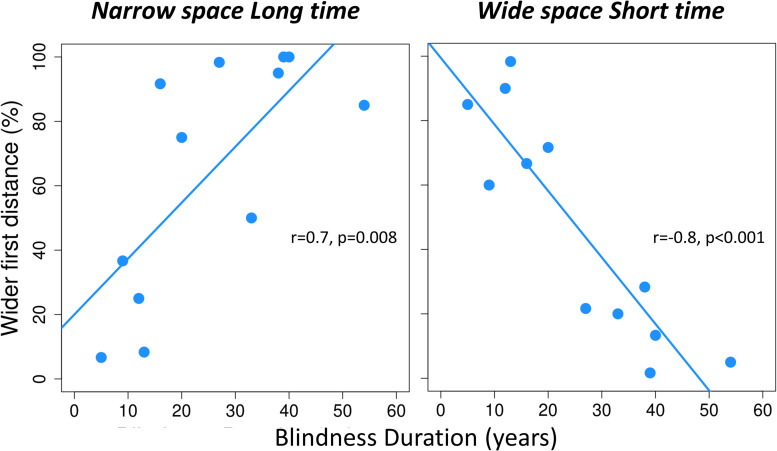
For each conflicting condition of the spatial bisection task, the perceived localization of S2 is plotted against years of blindness duration. For each conflicting condition (*narrowSpace_longTime, wideSpace_shortTime*), percentage of trials in which the first distance is reported as wider (i.e., perceiving S2 as played from the right) is plotted against years of blindness duration. Narrow and wide first spatial distances mean that S2 was played from the left (-4.5°) or right (+ 4.5°) side of participants respectively. Light blue lines indicate significant correlations. For each correlation, we report Pearson correlation coefficient (r) and significance level (p).

To investigate the impact of BD years on spatial bisection performance, we took into account each condition before merging for spatiotemporal coherence and conflict. The ANCOVA with percentage of wide first distance indicated as a dependent variable, condition as factor and BD years and age of onset as covariates, revealed a significant interaction between BD and condition [*F*(3, 32) = 11.51, *p* < 0.001]. No significant effect of age at onset emerged [for the main effect of onset: *F*(1, 32) = 0.4, *p* > 0.05; for the interaction between BD, condition and onset: *F*(3, 32) = 0.9, *p* > 0.05]. Given the significant interaction between BD and condition, we performed *post hoc* linear regressions between the logit-transformed percentage of trials in which the first distance was reported as wider and BD, separately for each condition. For coherent conditions (i.e., *narrowSpace_shortTime* and *wideSpace_longTime*), the percentage of wide first distance responses depended on the actual physical spatial position of the sound, which was congruent with the temporal delay. Thus, percentage of wide first distance was unrelated to BD years in the conditions *wideSpace_longTime* [*r* = -0.3, *p* > 0.05] and *narrowSpace_shortTime* [*r* = 0.02, *p* > 0.05]. However, for the conflicting conditions, the percentage of answer wide first distance responses was influenced by BD years. In the condition *narrowSpace_longTime* (*r* = 0.7, *p* = 0.01, Figure 3 left), LB individuals with shorter BD (i.e., fewer years of visual deprivation) answered based on the real spatial position of the stimulus (i.e., low percentage of wide first distance), despite the long temporal interval. With increasing BD years (i.e., more years of visual deprivation), LB individuals reported a higher percentage of wide first distances, although the first distance was narrow. Since the first temporal interval was longer, we can hypothesize that this result likely happens because the temporal coordinates of the stimulus trick LB participants with long BD. A similar pattern characterized responses in the other conflicting condition, *wideSpace_shortTime* (*r* = -0.8, *p* < 0.001, Figure 3 right). In this case, the slope of the relationship is reversed: with increasing BD years, the percentage of wide first distance answers decreases. Since the first temporal interval was shorter in this condition, we can again hypothesize that the lower percentage of wide first distance likely happens because the temporal coordinates of the stimulus trick LB participants with long BD. Therefore, individuals with long BD apparently tend to estimate the first spatial distance based on the time interval between the two stimuli.

### Impact of Years of Blindness on Neural Correlates of Spatial Bisection

To confirm behavioral findings, we investigated the effect of increasing years of BD on how the cortical response associated with spatial bisection skills is influenced by temporal information. Spatial bisection skills are reflected by an early (50–90 ms) ERP response in occipital areas contralateral to the second sound position in space (Campus et al., 2017), and also in early responses in temporal scalp sites (Gori et al., 2020b). Thus, we focused on the early (50–90 ms) activation after S2 in both occipital (O1, O2) and temporal (T7, T8) scalp sites. For each occipital (O1, O2) and temporal (T7, T8) electrode, a significant interaction between BD and condition was present for mean amplitude in the 50-90 ms time window after S2 [for O1: *F*(3, 32) = 29.25, *p* < 0.001; for O2: *F*(3, 32) = 19.29, *p* < 0.001; for T7: *F*(3, 32) = 14.4, *p* < 0.001; for T8: *F*(3, 32) = 16.04, *p* < 0.001]. The interaction between BD, condition, and age of onset was not significant [for O1: *F*(3, 32) = 1.54, *p* > 0.05; for O2: *F*(3, 32) = 0.54, *p* > 0.05; for T7: *F*(3, 32) = 1.34, *p* > 0.05; for T8: *F*(3, 32) = 1.79, *p* > 0.05], as well as the main effect of age of onset [for O1: *F*(1, 32) = 0.21, *p* > 0.05; for O2: *F*(1, 32) = 0.27, *p* > 0.05; for T7: *F*(1, 32) = 1.1, *p* > 0.05; for T8: *F*(1, 32) = 0.6, *p* > 0.05].

**FIGURE 4:**
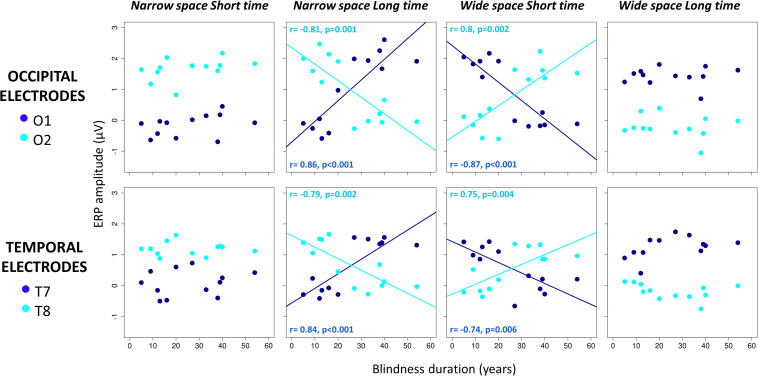
Correlations between years of blindness duration and mean ERP amplitude in occipital **(top)** and temporal **(bottom)** electrodes 50–90 ms after S2 of the spatial bisection task. For each participant and condition (*narrowSpace_shortTime, narrowSpace_longTime, wideSpace_shortTime, wideSpace_longTime*), ERP amplitude in O1/T7 (dark blue) and O2/T8 (cyan) is plotted against years of blindness duration. Lines indicate significant correlations. For each correlation, we report Pearson correlation coefficient (r) and significance level (p).

As expected, *post hoc* linear regressions indicated that ERP amplitude was not dependent on BD in coherent conditions. However, ERP amplitude was significantly related to BD years in conflicting conditions (see Figure 4). In the coherent condition *narrowSpace_shortTime*, ERP response in O2 and T8 is higher (see cyan dark blue dots in Figure 4), while ERP response in O1 and T8 is lower (see dark blue dots in Figure 4), regardless of BD (participants correctly perceived the sound as delivered from the left; for O1: *r* = 0.36, *p* > 0.05; for O2: *r* = 0.4, *p* > 0.05; for T7: *r* = 0.17, *p* > 0.05; for T8: *r* = 0.01, *p* > 0.05). Similarly, in the coherent condition *wideSpace_longTime*, ERP response in O1 and T7 is higher, while ERP response in O2 and T8 is lower, independent of BD (participants correctly perceived the sound as delivered from the right; for O1: *r* = 0.01, *p* > 0.05; for O2: *r* = 0.17, *p* > 0.05; for T7: *r* = 0.47, *p* > 0.05; for T8: *r* = 0.39, *p* > 0.05). In the coherent conditions the spatial and temporal contributions to the correct response are confounded. The conflicting conditions allow us to observe whether the response is aligned with spatial or temporal cues. In the conflicting condition *narrowSpace_longTime*, BD is negatively correlated with ERP amplitude in O2 (*r* = -0.81, *p* = 0.001) and T8 (*r* = -0.79, *p* = 0.002), and positively correlated with ERP amplitude in O1 (*r* = 0.86, *p* < 0.001) and T7 (*r* = 0.84, *p* < 0.001). In the conflicting condition *wideSpace_shortTime*, BD is negatively correlated with ERP amplitude in O1 (*r* = -0.87, *p* < 0.001) and T7 (*r* = -0.74, *p* = 0.006) and positively correlated with ERP amplitude in O2 (*r* = 0.8, *p* = 0.002) and T8 (*r* = 0.75, *p* = 0.004). Thus, the physical position of S2 still elicits a specific occipital and temporal contralateral response in LB subjects that recently lost sight, while after many years of BD the response becomes inverted and ipsilateral. The occipital and temporal sites of LB participants with short BD show activations similar to those found in the coherent conditions, contralateral with respect to the spatial position of S2. Responses of LB participants with long BD in the conflicting conditions are instead ipsilateral to the spatial position of S2. They are contralateral to the perceived virtual position of the sound based on its temporal delay.

**FIGURE 5:**
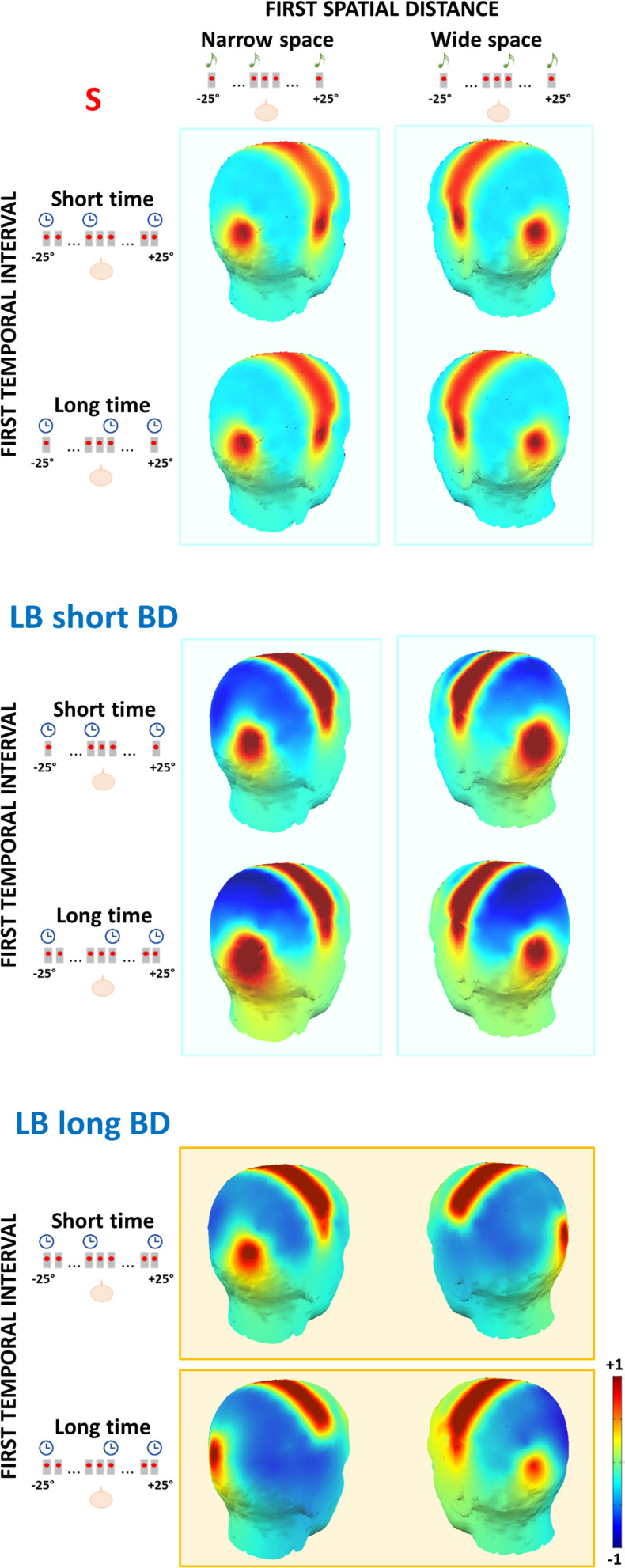
Topography of the mean ERP amplitude in the 50–90 ms time window after S2 of the spatial bisection task, for sighted (S), late blind people with short blindness duration (LB short BD) and late blind people with long blindness duration (LB long BD). The first spatial distance could be narrow (i.e., S2 played from -4.5°; **left panel**) or wide (i.e., S2 played from + 4.5°; **right panel**). The first temporal interval could be short (i.e., S2 played at -250 ms; first row for each group) or long (i.e., S2 played at + 250 ms; second row for each group). The contralateral occipital and temporal activation of sighted and LB with short BD individuals depends on the first spatial distance (i.e., vertical cyan rectangles). The same contralateral occipital and temporal activation in LB with long BD individuals depends on the first temporal interval (i.e., horizontal orange rectangles).

**FIGURE 6:**
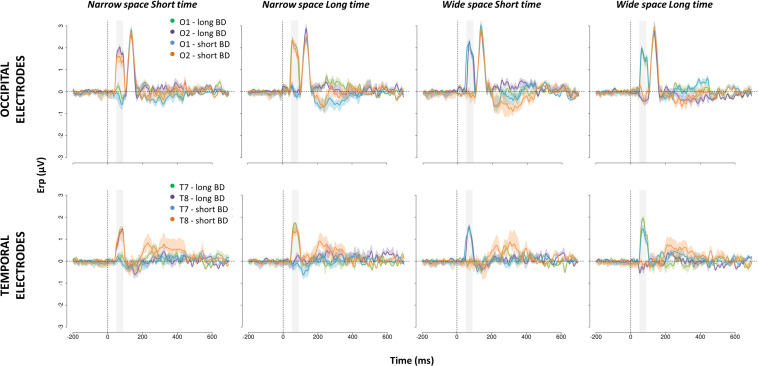
ERPs evoked in occipital **(top)** and temporal **(bottom)** scalp sites by S2 of the spatial bisection task. Waveforms (mean ± SEM) are reported for O1, O2, T7, and T8, separately for LB individual with long (i.e., long BD) and short (i.e., short BD) blindness duration. All coherent (i.e., *narrowSpace_shortTime*, *wideSpace_longTime*) and conflicting (i.e., *narrowSpace_longTime*, *wideSpace_shortTime*) conditions are displayed. In the conditions *narrowSpace_shortTime* and *narrowSpace_longTime* conditions, O1 and T7 are ipsilateral and O2 and T8 are contralateral to the physical location of the sound. In the conditions *wideSpace_longTime* and *wideSpace_shortTime* conditions, O1 and T7 are contralateral and O2 and T8 are ipsilateral to the physical location of the sound. On the *x*-axis, time = 0 ms represents the onset of the sound. The shaded area indicates the 50–90 ms time window.

To visualize these results, we arbitrarily divided the LB group into two subgroups based on BD median (short BD: BD < median BD, *N* = 6; long BD: BD > median BD; *N* = 6). In Figure 5, we report scalp maps of the mean ERP amplitude in the 50–90 ms time window after the S2 of the spatial bisection task, separately for each group (plus sighted individuals) and condition. Similarly, Figure 6 reports ERP waveforms elicited by S2 in the spatial bisection tasks in occipital (top) and temporal (bottom) scalp sites, separately for each condition and group.

In LB individuals with short BD, early occipital and temporal responses to S2 were high and lateralized based on S2 physical spatial position for both coherent and conflicting conditions (see Figure 5 center and Figure 6 blue and orange curves). The left occipital and temporal areas showed a response only when the stimulus was physically from the right side of the participant (i.e., *wideSpace_shortTime*, *wideSpace_longTime*) but not when it was from the left side (i.e., *narrowSpace_shortTime, narrowSpace_longTime*). Symmetrically, the right occipital and temporal areas responded when S2 was played from the left side, and did not respond when it was played from the right side. This pattern resembles that of the sighted group (Figure 5 top). However, in the long BD group, the same pattern was evident only for the coherent conditions. In coherent conditions, left occipital and temporal areas of LB individuals with long BD (Figure 5 bottom and Figure 6 green and violet curves) showed a similarly strong response when the sound was played from the right (*wideSpace_longTime*), and a similarly weak response when the sound was played from the left (*narrowSpace_shortTime*). Symmetrically, the right occipital and temporal areas showed an equally strong response when S2 was played from the left (*narrowSpace_shortTime*), and a similarly reduced activation when it was played from the right (*wideSpace_longTime*). In contrast, during conflicting conditions, LB individuals with long BD had topographically reversed responses compared to LB individuals with short BD. The response was contralateral with respect to the virtual position of the sound, suggested by its temporal coordinate as opposed to its real spatial location. Thus, for long BD subjects, a much stronger activation appeared in the left occipital and temporal areas when S2 was temporally closer to S3 (played from right) but physically played from the left of the subject (*narrowSpace_longTime*), and weaker when the sound was temporally closer to S1 (played from left) but physically played from the right (*wideSpace_shortTime*). Symmetrically, a stronger response emerged in the right occipital and temporal areas when S2 was temporally closer to S1 but physically played from the right side of the subject (*wideSpace_shortTime*), and weaker response was elicited when the sound was temporally closer to S3 but physically played from the left side (*narrowSpace_longTime*). These results suggest that years of blindness influence LB individuals to process spatial signals based on temporal properties.

The 50–90 ms time window is the first one that shows an effect associated with the spatial task. This is evident in Figure 6, showing the ERP waveforms elicited in occipital electrodes by S2. In agreement with our previous study (Gori et al., 2020b), a later P140 selective for S2, but not lateralized, appears in both groups independently of the condition. Typical auditory responses appear for all participants in central areas (see Supplementary Figure S1).

To exclude the possibility that our result is due to chronological age, we verified a lack of correlation between BD and chronological age for LB participants (*r* = 0.48, *p* > 0.05). Moreover, during the temporal bisection task performed as a control, cortical responses supported the specificity of temporal attraction during complex spatial representation following years of visual deprivation. In the temporal bisection task subjects were asked to evaluate timing presentations of sounds. Under this condition, all LB participants showed typical auditory responses and were not affected by the cross-domain conflict (i.e., only a response in central areas and a response in temporal areas contralateral with respect to the physical location of the stimulus were observed). In Supplementary Material, we report scalp maps in the selected time window (50–90 ms) after the S2 of the temporal bisection task (Supplementary Figure S2), and waveforms elicited by the stimulus in occipital (Supplementary Figure S3 top), temporal (Supplementary Figure S3 bottom) and central (Supplementary Figure S1 bottom) scalp sites. For details about waveforms and results in the sighted group, please refer to Gori et al. (2020b).

## Discussion

We previously showed that LB individuals with a long history of BD (i.e., > 25 years) do not show typical spatial bisection skills and neural correlates (Amadeo et al., 2019). Indeed, they do not show the early contralateral occipital activation associated with spatial bisection skills in sighted people and LB individuals who have recently lost vision. In this study, we investigated whether different performance and neural correlates of LB people with long BD are due to the use of an alternative strategy to represent space, based on temporal properties of stimuli. As expected, we demonstrate that years of visual deprivation following late blindness gradually lead complex spatial representations to be inferred based solely on temporal properties of events.

The spatial bisection task involves the evaluation of spatial distances among three stimuli, requiring relative comparisons between each pair of stimuli. To succeed at the task, people must understand Euclidean relationships and build sophisticated and well-calibrated auditory spatial maps. In agreement with previous studies (Campus et al., 2017; Amadeo et al., 2019), we confirm that sighted individuals and LB individuals with recent visual loss succeed at the spatial bisection task, and show a specific ERP response in occipital areas, likely involving the visual cortex, between 50-90 ms after the second of the three sounds of the task. The early occipital response is strong and contralateral to the physical spatial position of the second sound, which represents the first step in building a metric in space. More interestingly, we demonstrate that with increasing years of visual deprivation, performance and cortical activation become more influenced by temporal instead of spatial coordinates of the second sound. Indeed, the bisection task naturally combines spatial with temporal representations. The first and the third stimuli identify both a spatial distance and a temporal interval. By independently modulating the spatial and temporal coordinates of the second stimulus, it is possible to deliver signals coherent or conflicting in space and time. Thus, the bisection task allows us to investigate the weight given to spatial or temporal information in solving the task. To create coherent conditions, we associated a short spatial distance between the first and the second stimuli with a short temporal interval, and a wide spatial distance between the first and the second sound with a long temporal interval. To create conflicting conditions, we associated a narrow spatial distance between the first and the second sound with a longer temporal interval, and a wide spatial distance between the first and the second sound with a short temporal interval. A narrow spatial distance between the first and the second sound means that the second sound was physically played from the left side of the participant, while a wide spatial distance means that the second sound was physically played from the right side.

In coherent conditions, LB participants with long BD show a good performance and the same contralateral occipital response as sighted participants. Thus, they show a lateralized occipital activation of the visual cortex opposite to the actual spatial position of the second sound. Hence, by presenting coherent spatiotemporal information, we observe for the first time in LB people with long BD the early occipital response associated with spatial bisection skills. LB people with long BD displayed the response which was absent in our previous study that did not consider temporal information (Amadeo et al., 2019; Campus et al., 2019). Since there was no response when the temporal cues were not considered, and only spatial information was provided, we can suppose that the activation observed is related to the introduction of temporal information coherent with the spatial one. However, coherent conditions do not allow disentangling between the contributions of spatial and temporal information; this requires conflicting conditions. In conflicting conditions, with increasing BD in LB participants, performance and early cortical activation (i.e., 50–90 ms) differ. The response in long BD individuals recalls that of sighted people and blind participants with short BD but with an inverse topography. As BD years increase, left occipital activation emerges for long temporal intervals and narrow spatial distances (i.e., second sound physically played from the left but temporally closer to the sound from the right). Similarly, right occipital activation emerges for short temporal intervals and wide spatial distances (i.e., second sound physically played from the right but temporally closer to the sound from the left). These patterns of response also involve the temporal scalp sites of LB people with long BD.

Thus, our results suggest that occipital and temporal activation shows a lateralization pattern that aligns with the “temporal” position of the stimulus, determined by its temporal delay rather than its spatial coordinate. An impact of auditory temporal features on occipital areas has previously been observed in sighted people (Murray et al., 2016). For example, Bueti and Macaluso (2010) showed that temporal expectations of upcoming auditory events modulate activity in the occipital visual cortex, Romei et al. (2012) found that a single beep can phase-align alpha oscillations to sounds in the occipital cortex, and Cecere et al. (2015) demonstrated a link between an alpha frequency and the temporal window of the flash-beep illusion (Shams et al., 2000). Moreover, we have recently demonstrated that the visually evoked occipital component (C1) appears earlier during a temporal bisection of visual stimuli compared to a spatial bisection of the same stimuli (Amadeo et al., 2020). Within this context, Giard and Peronnet (1999) also revealed that visual cortex activates earlier the in response to synchronous audio-visual stimuli than visual stimuli alone. In the current experiment, the temporal information not only brings about a misperception of the stimulus in the occipital cortices, but also a sensory illusion which tricks the auditory processing at the early stages. In agreement with previous results (Gori et al., 2018, 2020b), the spatial nature of the visual cortices seems to be sufficiently dominant to drag the early activation of auditory cortices involved in the auditory processing of sounds. This is not the first study where sensory illusions are found to trick the early stages of processing of sensory cortices (e.g., Shams et al., 2001; Murray et al., 2002; Ress and Heeger, 2003; Watkins et al., 2006).

The behavioral performance confirms a temporal attraction during the spatial bisection task. Indeed, when we investigated the association between individual performance and years of visual deprivation, we observe that the higher the BD, the more the answer of participants was determined by the temporal cues in the conflicting conditions. These findings enrich previous findings, where we observed that following many years of visual deprivation, LB people gradually become less able to perform the spatial bisection task, and the associated early occipital response gets reduced and non-lateralized (Amadeo et al., 2019). Years of blindness drive to alternative ways of processing complex spatial representations, based on temporal instead of spatial information.

Our results (see Figures 5, 6), suggest that the LB group can be split in two; individuals having less than 20 years of blindness differed from individuals with more than 30 years of blindness. Unfortunately, we do not have enough participants aged 20–30 years old to fully understand the effect of BD on behavioral and neurophysiological parameters, particularly whether the influence is linear. A future experiment should specifically investigate what happens around 25 years after vision loss. Although it is the simplest method, the main reason we used linear regression analysis was for its robustness with respect to noisy or subsampled data. We further felt it unlikely that a drastic change occurs between these years, creating two independent groups. Moreover, other studies investigating LB people indicate years of visual deprivation tend to have a linear effect on certain parameters (Collignon et al., 2013; Wang et al., 2013; Tao et al., 2015). For example, Collignon et al. (2013) demonstrated that years of blindness of LB individuals are linearly associated with sound-related activity in some occipital regions. The effect of blindness duration on spatial representation that we observed is not due to aging. Indeed, in our experiment no association between years of blindness duration and chronological age was observed. In line with literature (Lepore et al., 2010; Li et al., 2013; Wang et al., 2013), we suggest that neural changes may be due to a progressive degenerative mechanisms following a lack of constant visual stimulation, such as structural atrophy and impairment of anatomical connections in the visual cortex.

It is important to mention that LB individuals represent a specific population, and it is difficult to find a large sample within this population without comorbidities or other confounding factors; therefore statistical power is necessarily affected by a small sample size. However, our control experiment confirms the specificity of the temporal attraction during the spatial representation. In the temporal bisection task, the cross-domain conflict only slightly affects the performance of LB participants, similarly to sighted people. Also, in line with our previous experiment (Amadeo et al., 2019), no peculiarities emerge at the cortical level in the conflicting and coherent conditions of the temporal bisection task. Moreover, the effect during the spatial task is not due to mere attention to space. The time window we focused on is very early (50–90 ms), and reflects more sensory rather than cognitive processing. Attention to space, in fact, is expected to weakly affect early ERPs, such as the occipital response of interest (Campus et al., 2017) and the N1 (Roder et al., 1999; Lange et al., 2006).

Results of this study add interesting insights into long-term neural plasticity. Since the construction of complex spatial representations is not compromised in late blind individuals with recent visual loss, visual experience during childhood seems to be important and sufficient for the complete development of complex spatial bisection skills and underlying neural circuits. In line with the cross-calibration theory (e.g., Burr and Gori, 2012), vision has time to solve its important role in calibrating complex auditory spatial representations during development. However, it is worth stressing that LB people with prolonged blindness show the same mechanism that characterizes the occipital and the temporal cortices of early blind people. Early blindness presents the same temporal focus during spatial evaluations in bisection tasks (Gori et al., 2018, 2020b). This suggests that strategies and neural circuits underlying the spatial bisection skills are strongly influenced by prolonged visual deprivation through long-term neural plasticity. Observing that prolonged visual deprivation later in life drives to the same reorganization of visual and auditory cortices as that seen in early visual deprivation provides essential cues about how the brain works. Our results stand against studies claiming that functional or structural reorganization is almost impossible beyond some critical periods (e.g., Cohen et al., 1999; Sadato et al., 2002; Noppeney, 2007). Instead, they are in line with literature showing that compensatory neural mechanisms can be adopted even later in life (e.g., Buchel, 1998; Burton, 2003; Voss et al., 2006; Collignon et al., 2013). However, most studies in this direction support the idea that auditory or tactile recruitment of occipital regions provide improved spatial skills, while our results seem to highlight some potential side effects of neuroplasticity. The auditory recruitment of the occipital brain in spatial bisection is not associated with better performance, but rather underlies an alternative way of building spatial representation.

On the one hand, using temporal information to infer spatial maps can be a useful strategy by which blind people can overcome problems in complex spatial representations. This strategy can be successful from time to time. There are situations in real life where spatial and temporal information is congruent, and this strategy would allow blind people to use unimpaired temporal coordinates to decode auditory spatial maps, facilitating interaction with others. On the other hand, this strategy could be dysfunctional when there is conflicting spatial and temporal information. There are real-life situations, such as accelerations or decelerations of environmental objects, where using temporal information to assume spatial positions would introduce a misperception of the stimulus, impacting one’s capability to interact with the environment. Therefore, our findings agree that there is no time window in which plastic changes can occur, cross-modal reorganization can occur even after the full development of the visual system. However, the direction of plastic neural changes is not apparent and does not always lead to successful behavioral outcomes.

Since the strategy adopted by early blind people and LB people with long BD is not always functional, one might wonder why the strategy exists and from where it came. According to the cross-calibration theory (Gori, 2015), during childhood the most reliable sense for a given perceptual property calibrates the other sensory modalities (see Gori et al., 2012). Within this framework, the visual modality, with its high spatial accuracy, is used to calibrate spatial representations of other senses. Similarly, the auditory modality, with its high temporal accuracy, is used to calibrate temporal representations of other senses. Sighted adults and LB individuals with short BD, who have experienced cross-sensory visual calibration during childhood, can build even complex spatial representation in the auditory modality. Early blind people, who did not experience visual calibration during childhood, instead focus on temporal cues (Gori et al., 2018, 2020b). Since after many years of visual deprivation, LB individuals also focus on temporal cues, the perceptual advantages of cross-sensory visual calibration seem to be gradually lost with the lack of visual experience. This result suggests that constant cross-sensory visual calibration may be necessary to maintain its beneficial effects. A hypothesis to explain temporal attraction during spatial representation involves considering the speed of the stimuli. It could be that, during development, the visual system calibrates the auditory sense of space by processing speed. When vision is available, the visual system may facilitate the transference of auditory processing from a temporal to a spatial coordinate system relying on speed processing. When visual inputs are absent, this transfer may not occur (i.e., in early blindness) or may gradually deteriorate (i.e., in late blindness), resulting in auditory maps based only on temporal cues for inferring complex spatial representations (Gori et al., 2020a). Thus, we speculate that, when visual-spatial networks are weakened by long-lasting lack of sensory stimulation, blind individuals assume constant velocity of environmental stimuli, thereby inferring space from time. This idea is supported by the Imputed Velocity Theory (Huang and Jones, 1982), which researchers previously proposed to explain the Tau and Kappa effects (Bill and Teft, 1972; Sarrazin et al., 2007; Kawabe et al., 2010). According to the latter, humans intuitively impute uniform motion to discontinuously displayed successive stimuli.

To conclude, in this work, we show that the long-lasting absence of visual input following late blindness leads to a reorganization of how people build complex spatial representations. After a long amount of time with no vision, a new strategy to represent space emerges, whereby the visual and auditory circuits use temporal information to interpret spatial metrics. Beyond theoretical relevance, the results of the current study have important repercussions for rehabilitation strategies following sensory loss. First, the knowledge that after prolonged blindness the effects of cross-sensory visual calibration are lost highlights the relevance of timing in interventions. People that become blind should be soon involved in early rehabilitation programs to activate compensatory strategies and not to lose perceptual-advantages from previous cross-sensory visual calibration. Long-term plasticity seems to lead to some maladaptive changes. We hypothesize that constant stimulation and training soon after vision loss may prevent them from occurring. For example, timely sensory-motor training such as the one previously validated in blind children (Finocchietti et al., 2015a) could have important effects. Secondly, if blind people benefit from spatiotemporal coherence, there is potential to develop new rehabilitation strategies by providing temporal cues to inform about spatial dimensions. For instance, trainings involving velocity tasks could be planned, where time can be used to infer space (as suggested in the model proposed by Gori et al., 2020a). Coherent temporal cues could be initially associated with spatial cues and then, only gradually, disassociated to promote recalibration. Blind people rely strongly on auditory information to orient themselves in their environments, and various techniques and approaches (e.g., serious games) could be realized to help them by taking advantage of temporal cues to learn about space.

## Data Availability Statement

The raw data supporting the conclusions of this article will be made available by the authors, without undue reservation, to any qualified researcher.

## Ethics Statement

The studies involving human participants were reviewed and approved by the Ethics Committee of the Liguria Region. The patients/participants provided their written informed consent to participate in this study. Written informed consent was obtained from the participant’s legal guardian/next of kin for the publication of any identifiable images or data presented in the study.

## Author Contributions

MG and CC conceived the study and designed the experiments. MA, MG, and CC carried out the experiments and analyzed the data, wrote the manuscript, prepared figures, and reviewed the manuscript. All authors contributed to the article and approved the submitted version.

## Conflict of Interest

The authors declare that the research was conducted in the absence of any commercial or financial relationships that could be construed as a potential conflict of interest.
