# Supplementary material for: Years of Blindness Lead to “Visualize” Space Through Time
Source: Front Neurosci. 2020 Aug 4;14:812. doi: 10.3389/fnins.2020.00812 (PMC7418563; doi:10.3389/fnins.2020.00812)
Supplement: Supplementary file 1 [file Data_Sheet_1.pdf]

Supplementary material

Years of blindness lead to "visualize" space through time

Maria Bianca Amadeo[1,2], Claudio Campus[1], Monica Gori[1]

[1] Unit for Visually Impaired People, Istituto Italiano di Tecnologia, Genova, Italy
[2] Università degli studi di Genova, Department of Informatics, Bioengineering, Robotics and Systems Engineering, Genova, Italy

*Correspondence: mariabianca.amadeo@iit.it, Fondazione Istituto Italiano di Tecnologia, Via Melen, 83 - 16152 Genova (Italy).

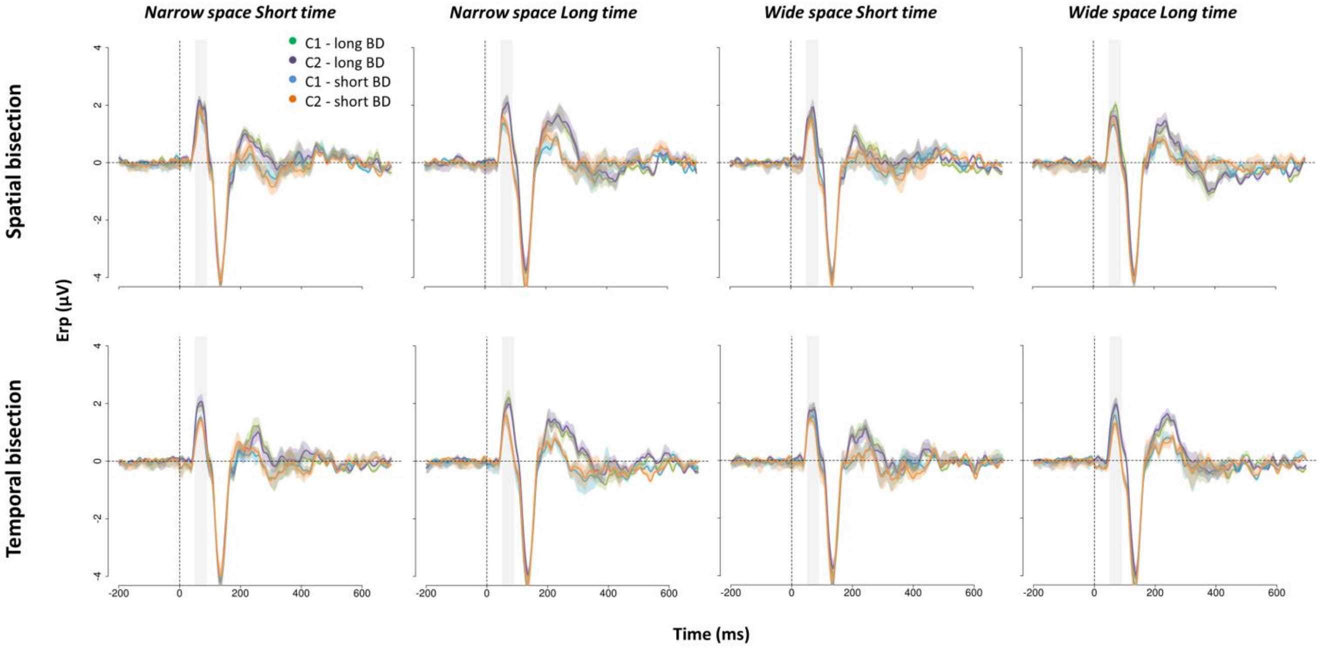

Fig. S1 ERPs (mean±SEM) elicited in central electrodes by S2 during spatial (top) and temporal (bottom) bisection task. Both left (C1) and right (C2) electrodes are reported for late blind (LB) people with long (long BD) and short (short BD) blindness duration. Each coherent (i.e. *NarrowSpace_shortTime*, *WideSpace_longTime*) and conflicting (i.e. *NarrowSpace_longTime*, *WideSpace_shortTime*) condition is considered separately. On the x-axis, t=0 is sound onset. The shaded area delimits the selected time window (50–90 ms).

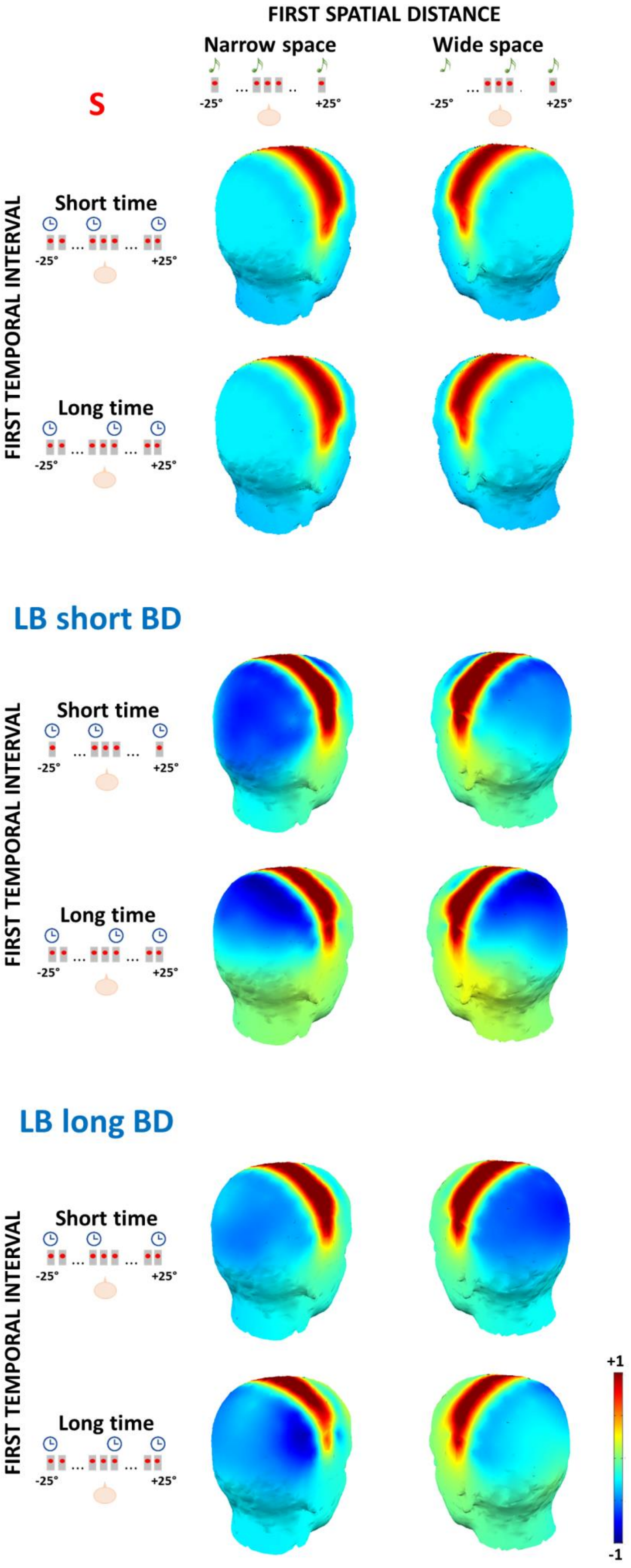

Figure S2. Scalp maps of the mean ERP amplitude in the selected (50-90 ms) time window after S2 of the temporal bisection task, for sighted (S), late blind people with short blindness duration (LB short BD) and late blind people with long blindness duration (LB long BD). The first spatial distance could be either narrow (i.e. S2 delivered from -4.5°; left panel) or wide (i.e. S2 delivered from +4.5°; right panel), and the first temporal interval could be either short (i.e. S2 delivered at -250 ms; first row for each group) or long (i.e. S2 delivered at +250 ms; second row for each group). For all participants, sounds elicited typical auditory response in central and temporal areas, idependentely of the condition.

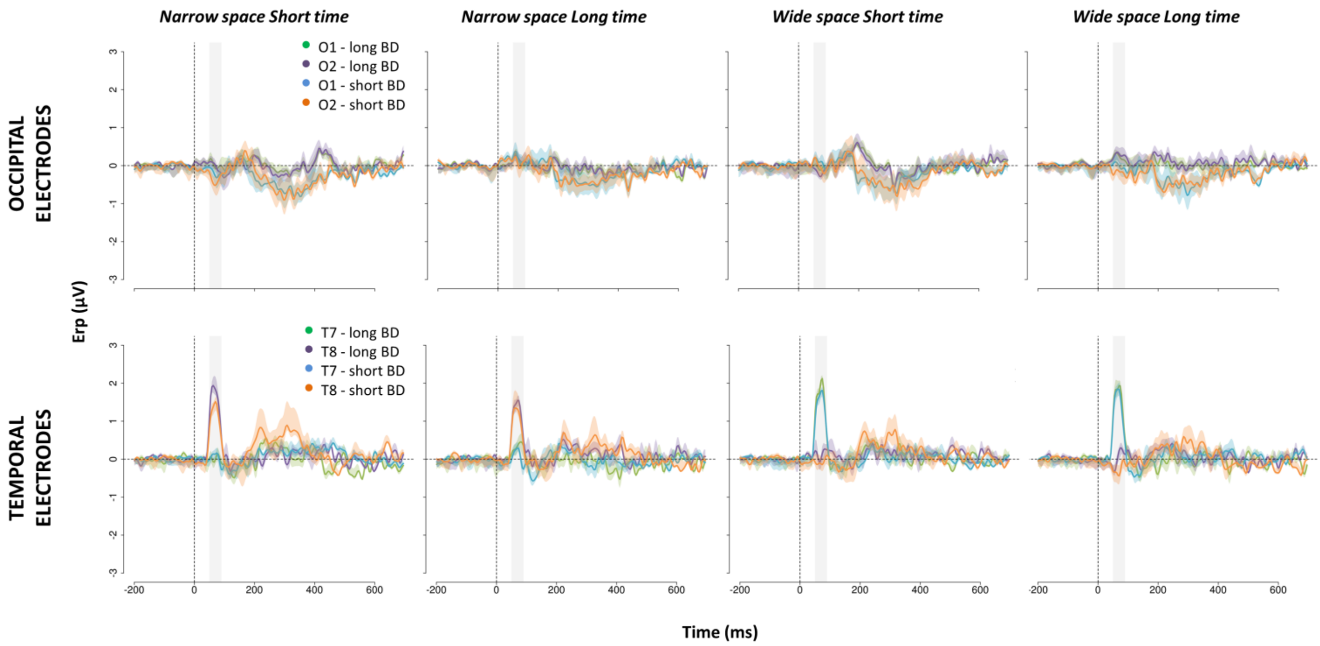

Fig. S3 ERPs (mean±SEM) elicited by S2 during the temporal bisection task in occipital (top) and temporal (bottom) electrodes. Both left (O1, T7) and right (O2, T8) electrodes are reported for late blind people with short blindness duration (LB short BD) and late blind people with long blindness duration (LB long BD). Each coherent (i.e. *NarrowSpace_shortTime*, *WideSpace_longTime*) and conflicting (i.e. *NarrowSpace_longTime*, *WideSpace_shortTime*) condition is considered separately. On the x-axis, t=0 is sound onset. The shaded area delimits the selected time window (50–90 ms).
